# Spatial and temporal analysis of road traffic crashes and ambulance responses in Lagos state, Nigeria

**DOI:** 10.1186/s12889-023-16996-8

**Published:** 2023-11-17

**Authors:** Aina Olufemi Odusola, Dohyo Jeong, Chenchita Malolan, Dohyeong Kim, Chinmayee Venkatraman, Olusegun Kola-Korolo, Olajide Idris, Oluwole Olayemi Olaomi, Fiemu E. Nwariaku

**Affiliations:** 1 Department of Community Health & Primary Health Care, Lagos State University Teaching Hospital, 1—5, Oba Akinjobi Road, Ikeja, Lagos Nigeria; 2 School of Economic, Political, and Policy Science, University of Texas at Dallas, Richardson, TX USA; 3 Department of Surgery, Office of Global Health, University of Texas Southwestern Medical Center, 5323 Harry Hines Boulevard, Dallas, TX 75390 USA; 4 Lagos State Ministry of Health, Block 4, The Lagos State Government Secretariat Complex, Alausa, Lagos, Ikeja Nigeria; 5 Department of Surgery, Central Business District, FCT, National Trauma Centre, National Hospital Abuja, Plot 321, Abuja, Nigeria; 6 Department of Surgery, Center for Global Surgery, University of Utah, 30 N 1900 E, Salt Lake City, Utah 3B110 USA

**Keywords:** Road crashes, Road traffic injuries, Pre-hospital care, Geospatial analysis, Resource Planning, Lagos state

## Abstract

**Background:**

Sub-Saharan African countries, Nigeria inclusive, are constrained by grossly limited access to quality pre-hospital trauma care services (PTCS). Findings from pragmatic approaches that explore spatial and temporal trends of past road crashes can inform novel interventions. To improve access to PTCS and reduce burden of road traffic injuries we explored geospatial trends of past emergency responses to road traffic crashes (RTCs) by Lagos State Ambulance Service (LASAMBUS), assessed efficiency of responses, and outcomes of interventions by local government areas (LGAs) of crash.

**Methods:**

Using descriptive cross-sectional design and REDcap we explored pre-hospital care data of 1220 crash victims documented on LASAMBUS intervention forms from December 2017 to May 2018. We analyzed trends in days and times of calls, demographics of victims, locations of crashes and causes of delayed emergency responses. Assisted with STATA 16 and ArcGIS pro we conducted descriptive statistics and mapping of crash metrics including spatial and temporal relationships between times of the day, seasons of year, and crash LGA population density versus RTCs incidence. Descriptive analysis and mapping were used to assess relationships between ‘Causes of Delayed response’ and respective crash LGAs, and between Response Times and crash LGAs.

**Results:**

Incidences of RTCs were highest across peak commuting hours (07:00-12:59 and 13:00-18:59), rainy season and harmattan (foggy) months, and densely populated LGAs. Five urban LGAs accounted for over half of RTCs distributions: Eti-Osa (14.7%), Ikeja (14.4%), Kosofe (9.9%), Ikorodu (9.7%), and Alimosho (6.6%). On intervention forms with a Cause of Delay, Traffic Congestion (60%), and Poor Description (17.8%), had associations with LGA distribution. Two densely populated urban LGAs, Agege and Apapa were significantly associated with Traffic Congestion as a Cause of Delay. LASAMBUS was able to address crash in only 502 (36.8%) of the 1220 interventions. Other notable outcomes include: No Crash (false calls) (26.6%), and Crash Already Addressed (22.17%).

**Conclusions:**

Geospatial analysis of past road crashes in Lagos state offered key insights into spatial and temporal trends of RTCs across LGAs, and identified operational constraints of state-organized PTCS and factors associated with delayed emergency responses. Findings can inform programmatic interventions to improve trauma care outcomes.

**Supplementary Information:**

The online version contains supplementary material available at 10.1186/s12889-023-16996-8.

## Background

Globally, Road Traffic Crashes (RTCs) are a dreadful public health hazard. RTCs cause serious road traffic injuries (RTIs) and associated potential fatal outcomes, or permanent disabilities among survivors. World Health Organization (WHO) estimates that 1.35 million of the 5.8 million annual injury related deaths are attributed to RTIs [1]. Of this, about 90% occur in low- and middle-income countries (LMICs). Yet another 20 to 50 million survivors suffer nonfatal permanent disabilities [1]. RTIs cost most countries significant job losses, more than 3% of their Gross Domestic Product, and reduced earnings from permanent disabilities [1]. Compared to high income countries LMICs carry a disproportionately higher percentage of global RTI burden. For example in 2019 out of 100,000 populations, Nigeria (20.75 persons) and Ghana (25.67 persons) reported disproportionately higher RTI mortality than high income countries like Germany (3.78 persons) and United Kingdom (3.21 persons) [2].

With projected population of 198,387,623 in 2018 and 223,804,632 in 2023, Nigeria is the most populous Black Country in the world [3], and Lagos is her most densely populated state (6710 population per Km^2^) [4]. As in many LMICs Nigeria inclusive, multiple factors contribute to high RTI burden. These include managerial, infrastructural and environmental constraints like overcrowded neighborhoods, poor road infrastructure, poor road networks, unsafe vehicles, adverse weather conditions, inadequate capacity for post-crash care and inefficient traffic management capacity. Other constraints include behavioral RTI risk factors like phone driving, drink driving, over-speeding, non-use of car seat belts, child restraints and motorcycle crash helmets by commuters, disregard for Zebra crossings and traffic lights by drivers, and non-use of pedestrian bridges and side-walks by pedestrians [1, 5].

Furthermore, Lagos is prone to seasonal variations with periodically adverse weather conditions associated with Harmattan fogs, heavy rains and attendant poor visibility and road flooding making driving hazardous and increasing potential for RTCs. The high population density of Lagos predisposes the state to heavy human and vehicular traffics and high risk for RTCs at particular geographic locations during Nigeria’s many cultural festivities and holiday seasons [6–8]. Over the last two decades, concerned about the high burden of RTIs in the state successive governments have implemented new road safety measures including: 1) establishing the Lagos State Ambulance Service (LASAMBUS) in 2001 to among others strengthen post-crash care; 2) increasing capacity of Lagos State Emergency Medical Services to improve in-hospital trauma care; 3) establishing Lagos State Traffic Management Authority to improve traffic control efficiency; and 4) strengthening capacity of the existing Public Work Department to improve and maintain the state’s road infrastructure. It is possible to use information from hotspots analysis of locations and times of past road crashes and explore spatial and temporal dimensions of interfaces between nature, human populations and the environment to drive improvements in pre-hospital trauma care outcomes. Such information can guide interventions to improve traffic management efficiency and road safety.

In recent times, it has become increasingly possible to predict spatial and temporal dimensions of road traffic crash hotspots and associated fatalities and injuries at multiple locations using Geographic Information Systems (GIS) [9]. Based on methods of spatial statistical analysis, various models can be built into GIS to more accurately predict RTCs hotspots [10, 11] and improve resource allocation planning for better management of road crashes and associated injuries. For example in Baltimore city USA, geospatial analysis of road crashes from 2009 to 2013 highlighted the important roles played by seasonality in incidence of road crashes. The researchers suggested a deeper analysis of the relationships between RTC risk factors like seasonal variations and geospatial characteristics of road crashes to tailor proposed interventions more efficiently [12]. In a similar vein, Soltani and Askari (2017) explored spatial auto-correlation of past road crashes in Iran to inform better strategies for traffic safety planning and management [13].

Several other studies from Nigeria and other LMICs also highlighted usefulness of analyzing spatial and temporal information on past road crashes to determine severity index of crashes and identify locations most prone to crashes wherefore resources and attention could be judiciously directed [14–21]. In planning interventions to reduce road crashes the variations observed in daily peaks of crashes can provide actionable information to predict and proactively manage traffic congestions at road crash hotspots. For example in Miami-Dade County in USA, researchers effectively used spatio-temporal analytic approach to examine the roles played by time-of-day, day-of-week and seasonality in the distribution of traffic crashes and determination of crash hotspots [22].

In this study, we explored spatial and temporal trends of past road crashes across 20 urban and rural Local Government Areas (LGAs) of Lagos state over six months (December 2017 – May 2018), and used the analyzed information to: 1) highlight geospatial dimensions of the RTCs managed by LASAMBUS; 2) evaluate outcomes of LASAMBUS interventions by LGA; and 3) assess efficiency of LASAMBUS response by LGA. Findings have potential to drive improvement in traffic management efficiency and judicious allocation of scarce PTCS resources to neediest locations and most auspicious times and seasons.

### Lagos state ambulance service

Lagos State Ambulance Service (LASAMBUS) was established in 2001 to provide pre-hospital emergency and trauma care services in Lagos state [23]. At the time of the current study LASAMBUS operates with 25 ambulances and crews from 25 ambulance points distributed fairly evenly across urban and rural metropolis of Lagos state. In practice, the activities of a subset of five of the 25 ambulances are coordinated through larger operational bases (OB) of each of the five subset of ambulances. There are five such operational bases, each located within the five administrative divisions of Lagos state (Ikorodu, Badagry, Ikeja, Lagos Island and Epe). Procedurally LASAMBUS operates day-time schedules from 8am to 5 pm and night-time schedules from 5 pm to 8am next day. For logistics and security reasons, each day-time ambulance team operates from within one of the five administrative divisions and return to Base at 5 pm to end the day-time operations. Each night-time ambulance team similarly operates within one of the five divisional Bases from 5 pm to 8am next morning to end night-time operations. But LASAMBUS encountered prevailing operational constraints which allowed only one ambulance team per OB to operate during night-time shifts compared to five teams that operate during day-time shifts. This implies that one night-time ambulance crew has to cover much larger geographic area within each administrative division compared to smaller area covered by each day-time ambulance crew.

In practice, immediately an in-coming emergency call is received at the Call Center the nearest ambulance team is dispatched to the crash site by the center’s dispatcher unit. The period from the time the call center receives an emergency call to the time the dispatched ambulance arrives at the crash site is recorded as emergency response time (ERT). LASAMBUS currently has an average response time of 17.0 min. (7–60 min.) [24] and a goal to reduce this to 12 min in the nearest future. For obvious reasons, day-time ERTs are usually shorter than night-time ERTs because night-time ambulances operate from within much larger operational area of an entire Divisional Base and therefore cover longer distances to reach potential crash sites compared to the day-time ambulances which operate within much smaller areas defined by the small operational radius of each of five ambulance points. During emergency response operations LASAMBUS crew manually documents clinical, temporal and spatial records of care for each crash victim on intervention forms.

## Methods

### Data collection

Using descriptive cross-sectional design we collected and analyzed data on emergency responses to past road crashes involving 1352 victims as documented on intervention forms from December 2017 to May 2018. Each completed intervention form has records of emergency response times, crash dates and periods, crash locations, critical care parameters of victims and other care related information including initiators of emergency call, attending ambulance crew, specific accident scenario or type, body injury site and severity, transfer destinations, treatment intervention and monitoring, trauma prompts, triage trauma score, causes of delayed response and patient demographics.

### Context and setting

We studied all documented road crashes across the old 20 Local Government Areas (LGAs) of Lagos state. In 2018, Lagos State had a projected population of 12,531,530 [25]. The State is located in South–West, Nigeria, on the Bight of Benin on longitude 20 42’E and 32 2’E, and latitude 60 22’N and 60 2’N respectively. The state is bounded in the North and East by Ogun State, in the West by Republic of Benin, and in the south by the Atlantic Ocean. Its territorial extent encompasses an area of 358,862 hectares or 3,577 sq. km. The communities in Lagos State make up the 20 LGAs including 4 sparsely populated rural LGAs, and 16 densely populated urban LGAs. For political reasons and ease of administration the state was later divided into five large administrative divisions comprising Ikeja, Badagry, Ikorodu, Lagos Island and Epe while 37 new political districts designated as Local Council Development Areas (LCDAs) were carved out of the existing (old) 20 LGAs to make up 57 new political structures comprising 20 new LGAs and 37 new LCDAs [26]. Spread across the state are numerous public health institutions and facilities which provide healthcare services to citizens across urban and rural parts of the state, including 2 Tertiary (Teaching) Hospitals, 4 Specialist Hospitals, 26 General Hospitals and 7 Comprehensive Health Centers [26]. In addition, there are over 320 smaller capacity Primary Health Centers spread across the state.

We observed that many parts of Lagos metropolis are largely overcrowded with new housing units and estates increasingly developed and spilling over into neighboring states while many towns and city centers easily become characterized by high volume vehicular traffics and poorly developed road infrastructure.

**Fig. 1:**
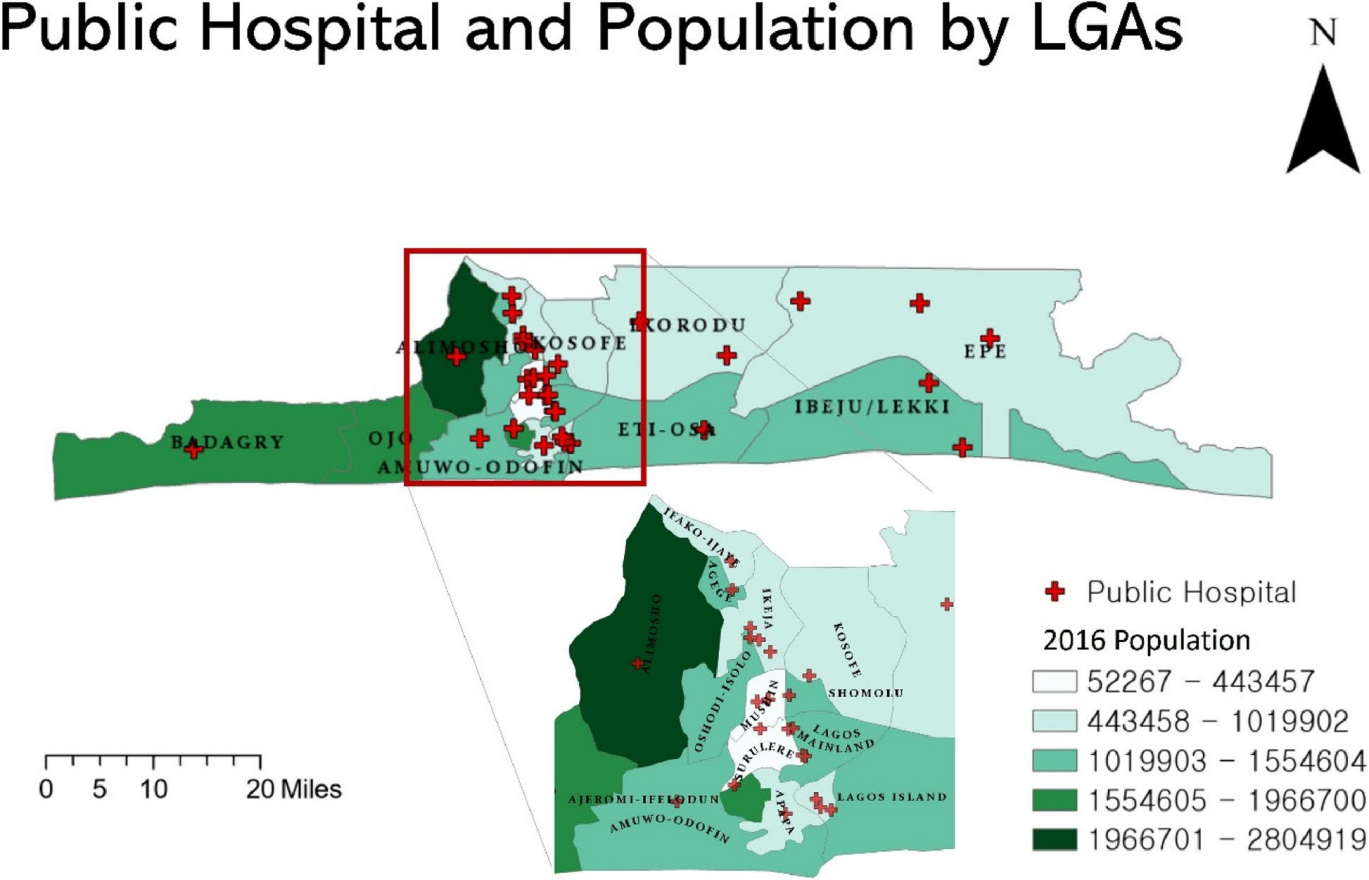
Population and Public hospital distribution by LGAs

Figure 1 shows the distribution of population of Lagos State in 2016 and public hospitals for each LGAs. The population figures were based on data released by the Lagos State Government (About Lagos. https://lagosstate.gov.ng/about-lagos/), and the distribution of public hospitals was based on data from the NIGERIA Health Facility Registry (https://hfr.health.gov.ng/). The population is concentrated mainly in the central and western parts of Lagos. The most central part is relatively sparsely populated while hospitals are concentrated in the center of the state.

Table 1 below shows the number of people in each district of Lagos State and the number of public hospitals in that district. First, Alimosho and Mushin districts have high population numbers, each with two public hospitals. Meanwhile, Lagos Mainland has a relatively low population although seven public hospitals operate there. On the other hand, the Ojo region has a large population but no public hospitals. Lagos Island is home to five public hospitals despite its relatively small area.

**Table 1:** Distribution of population and public hospitals by LGA

LGAs	2016 Population	Number of Public Hospitals
Agege	1,415,547	1
Ajeromi Ifelodun	1,966,700	2
Alimosho	2,804,919	1
Amuwo Odofin	719,337	1
Apapa	715,792	1
Badagry	521,267	1
Epe	443,457	3
Eti Osa	1,347,653	2
Ibeju Lekki	136,394	1
Ifako Ijaiye	1,019,902	1
Ikeja	888,903	3
Ikorodu	944,158	2
Kosofe	1,280,646	1
Lagos Island	1,178,200	5
Lagos Mainland	862,524	7
Mushin	1,810,797	2
Ojo	1,290,113	0
Oshodi Isolo	1,554,604	2
Shomolu	1,404,666	1
Surulere	1,746,183	2

### Data management and analysis

We used descriptive analysis and mapping to evaluate relationships among variables. An electronic version of the intervention form was created on REDcap to explore the statistical relationships among specific indicators such as date of call, time of call, demographics of victim, geographic distribution of crash, causes of delayed response and information from the narrative remarks section. The initial dataset of 1352 however showed varying degrees of missing information for specific data fields, such as 'Response time' and 'Address of collision location.' Consequently, this study effectively utilized 1220 out of 1352 cases, after excluding data with missing response times or missing accident locations from the initial dataset of 1352. Specifically, the percentage of omitted response times for ambulances was 5.8%, and the rate of missing accident locations was 20.3%. The former (omitted response times) was addressed using a pair-wise deletion technique (see Additional file 1) to account for observations that recorded only ‘time call received’ or only ‘time crew arrived at crash site’ for which we could therefore not calculate response time. We defined Response Time as the time between when LASAMBUS received an emergency call and when the dispatched crew arrived at crash scene, and we were able to calculate ‘response times’ in 90.2% of the observations. The missing information on crash location addresses was addressed by engaging knowledgeable research assistants including LASAMBUS drivers and crew members who are very familiar with specific address locations across the state to identify crash locations on forms with incomplete records. Assisted with a broad street map of Lagos which includes all 20 LGAs, the research assistants were able to locate most of the incompletely recorded addresses in specific streets or LGAs of concern to provide the missing information.

Furthermore, using five response categories we reviewed narratives in the remarks section on each form to determine ‘Outcome of Calls’ as follows: (I) Addressed Crash, meaning crash was found and addressed by LASAMBUS; (II) No Crash (False Call), meaning LASAMBUS located the reported crash site and found no crash; (III) Crash Already Addressed, meaning crash victim was already cared for or evacuated by other interventionists by the time LASAMBUS arrived site; (IV) Did Not Respond, meaning for logistics or other constraints, LASAMBUS could not respond to the call; and (V) Other, meaning few other scenarios like security challenges or communal disturbances in areas of crash. To determine total number of ‘Causes of Delay’ from all included intervention forms we noted the listed ‘Causes of Delay’ including poor access, traffic congestion, community disturbance, weather, poor description, proximity and faulty ambulance that were not appropriately marked as expected on indicated sections of some forms, but were nonetheless mentioned as narrative in the “Remarks” section. We therefore added the Outcomes section to the REDcap electronic form created for analysis and added a second Trauma Prompts section, and a second Cause for Delay section and supplemented this with information from the narrative remarks section to account for specific missing information on some incompletely filled forms. Therefore, to determine geospatial distribution of crashes relative to their respective LGAs we analyzed the addresses on the forms and discovered that 1,220 (90.2%) of the 1,352 forms were documented and specifically located within concerned LGAs. Assisted with STATA 16 and ArcGIS pro we conducted descriptive analysis and mapping to evaluate the relationship between ‘Causes of Delay’ and respective LGAs. Using descriptive analysis we explored spatial and temporal relationships between times and locations of crashes including: operational periods (day-time, night-time) vs. incidence of RTCs; seasonal changes (dry, rain and harmattan) vs. incidence of RTCs; festivity period (Christmas, Ramadan and Easter) vs. incidence of RTCs; and population density of crash LGA vs. incidence of RTCs.

### Ethical considerations

Citing the U.S. Department of Health & Human Services’ regulation 45 CFR 46.102, in March 2019 the Institutional Review Board of University of Texas Southwestern Medical Center from where the research project originated, approved the study as non regulated research. Furthermore researchers followed the Helsinki declaration principles and ethical conduct of observational research as outlined in STROBE guideline.

## Results

**Fig. 2:**
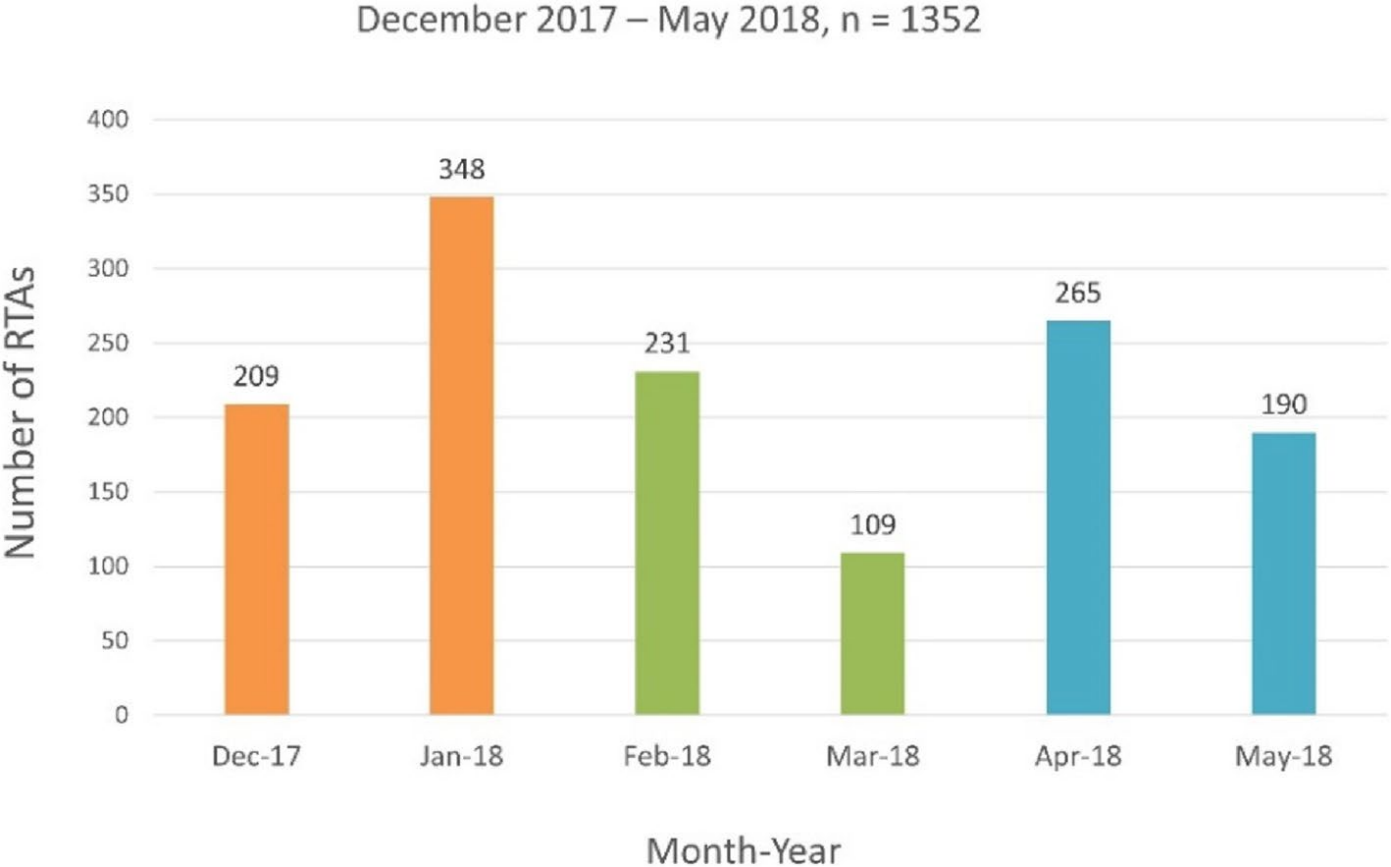
Descriptive statistics: number of RTCs (intervention forms) by month

We analyzed data from 1220 intervention forms spanning December 2017 to May 2018 (Fig. 2). The Median age of crash victims was 35 years. Most participants (71.3%) were males. The median Response Time was 20.0 min. Most emergency calls (56.7%) were attended to during Night-time operations (5pm – 8am) compared to much fewer calls attended to during Day-time operations (8am – 5pm), (43.3%). Compared to fewer operations (31.1%) that were implemented during the month of April and May (Rainy season), much more operations (41.9%) took place during December and January combined (harmattan, foggy). Our data shows that crashes for some months with significant religious and cultural festivals are higher than for other months. This is not surprising as there is increased potential for commuting and traveling within and across the state during religious and cultural festivities when people travel to other towns, villages or states. This appeared to influence road traffic crashes (RTCs) trends and incidences as many crashes were attended to during Christmas celebration in December (16%), and Easter and Ramadan celebrations in April (20.4%). The influence of adverse seasonal conditions on the incidence of crashes was manifest from December to March (Harmattan and dry season) during which driving tended to be hazardous and the potential for crashes increased due to poor visibility from hazy fogs.

The analysis of outcomes of interventions showed that LASAMBUS was able to attend to crash victims in just 449 (36.8%) road crashes out of the 1220 calls received. The remaining calls were not effectively executed for various reasons including: No crash (false calls), 26.6%; Crash was already addressed by the time LASAMBUS got to crash scene, 22.1%; LASAMBUS could not respond to the calls due to logistic constraints, 1.4%; and other miscellaneous reasons like ‘victim already died’ and ‘victim found but no injuries’, 13%. The relationship between geographic characteristics of crash locations and crash incidences across LGAs was manifest in the dataset; we observed that most crashes were attended to in densely populated urban LGAs (55.2%) compared to fewer attendances in sparsely populated rural LGAs. It was therefore no coincidence that with exception of Ikorodu LGA, the four other LGAs with frequent crash calls are located within densely populated urban areas. Furthermore, just five out of twenty LGAs accounted for over half (55.2%) of the RTCs distribution: Eti-Osa (14.7%), Ikeja (14.4%), Kosofe (9.9%), Ikorodu (9.7%), and Alimosho (6.6%). In the five LGAs with frequent calls, Outcome II (No Crash or False Call) and Outcome III (Crash Already Addressed) accounted for 26.4% and 24% of the total RTCs burden. We also observed a variance in the number of RTCs with a specific outcome and the proportion of RTCs with that same outcome within the LGA. For example, Eti-Osa had the highest number of RTCs with Outcome II (No Crash or False Call) whereas Ojo at 25.6% had the highest proportion of RTCs with the same outcome. This pattern was equally seen with Outcome III (Crash Already Addressed).

We found that two densely populated urban LGAs, Agege and Apapa were significantly associated with Traffic Congestion as a Cause of Delay. There were, however, too few observations to warrant further assessment of the relationship between Poor Access and the LGAs. Nevertheless, analysis of response times across LGAs showed significant variations; LGAs in which majority of RTCs occurred had lower average response times than others. Indeed Eti-Osa (21.3 mins.), Ikeja (19.2 mins.), and Kosofe (19.8 mins.) recorded three of the lowest LASAMBUS response times. Interestingly analysis of response time’s distribution by LGA showed that none of the three aforementioned LGAs were significantly associated with response times. In contrast those LGAs with higher response times such as Agege (33.2 mins.) and Alimosho (38.9 mins.) were significantly associated with response times.

Regional traffic accidents in Nigeria are generally impacted by seasons and time of the day [27]. However, it remains unclear whether the Lagos State Ambulance Service’s RTCs and Response Time follow the same pattern, or if there are specific local differences.

First, this study looked for the difference between RTCs and Response Time (RT) according to the season in LGA units (Fig. 2). In this study, traffic accident patterns in the dry and rainy seasons, the main seasons in Nigeria, were identified, and accident patterns that occurred according to the changing seasons were analyzed by dividing the seasons into Harmattan (foggy) (December and January) and Dry (February and March) and the Rainy season (April, May). In particular, Harmattan (foggy) has been pointed out as a cause of various accidents, including road traffic crashes in Nigeria [28–30]. 

**Fig. 3:**
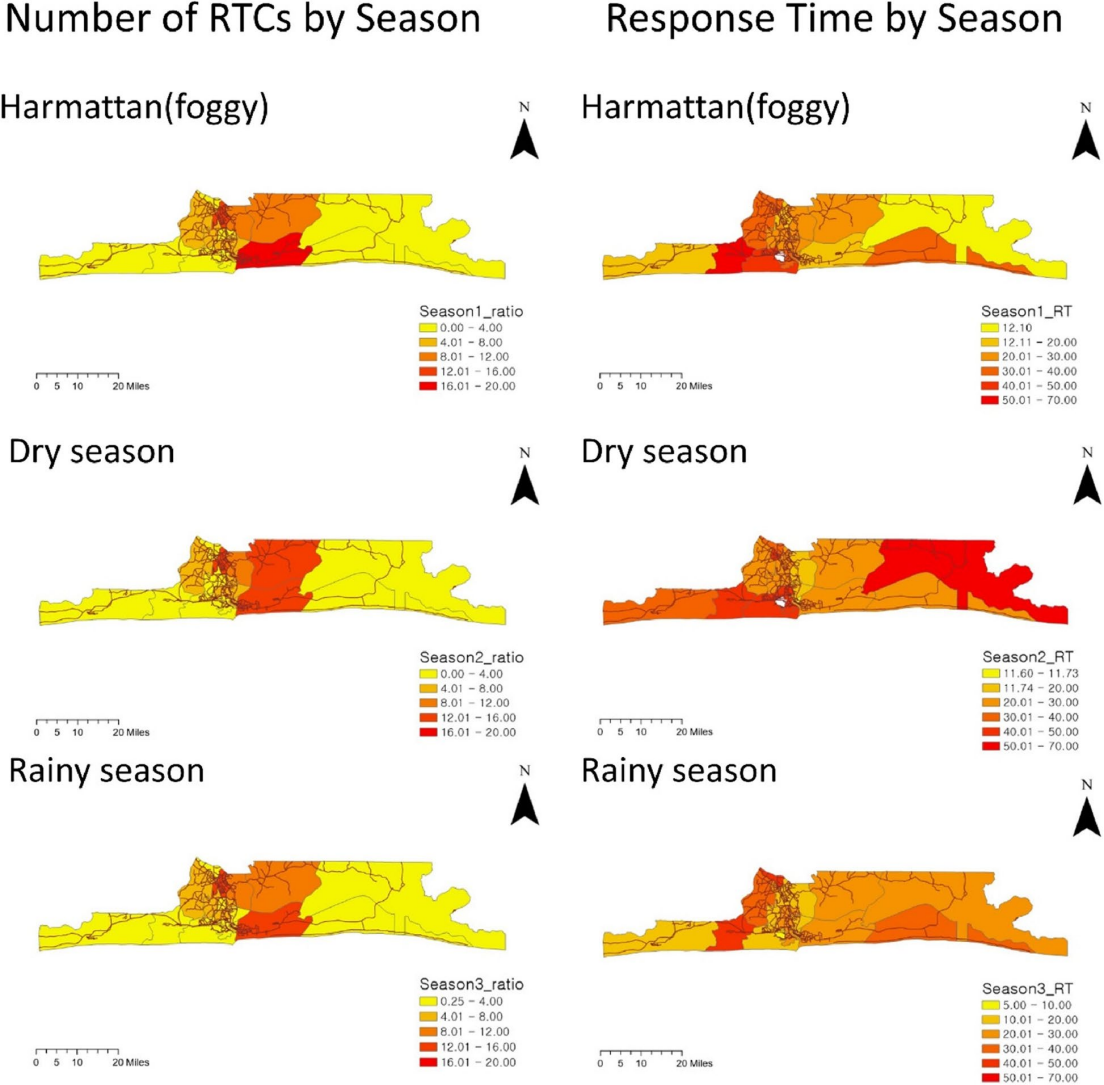
Number of RTCs and response time (RT) by season in Lagos LGAs

Figure 3 presents the road traffic crashes (RTCs) ratios (left column) and response times (right column) of each region by season. The incidence of RTCs was highest in the central region (Ikorodu, Eti-Osa, and Ikeja), with lower rates in outlying regions. However, regional differences in RTCs rates were consistent across seasons, with specific regions having consistently high rates. In contrast, the patterns of response times varied by region and season. During the Harmattan (foggy) season, several LGAs, such as Ojo and Amuwo-Odofin, had low RTC numbers but relatively high response times. The response time of Ojo was significantly impacted by the Harmattan (foggy) season, while the response times of Epe, Badagry, and Eti-Osa were not significantly affected. In contrast, during the dry and rainy seasons, Epe's response time increased rapidly, while Ojo's response time decreased. Each LGA exhibited a consistent pattern of RTCs for each season, but response times varied by season.

Next, to compare RTC rates and response times by time period, this study analyzed four time periods: Dawn (00:00-06:59), Morning (07:00-12:59), Afternoon (13:00-18:59), and Night (19:00-23:59).

**Fig. 4:**
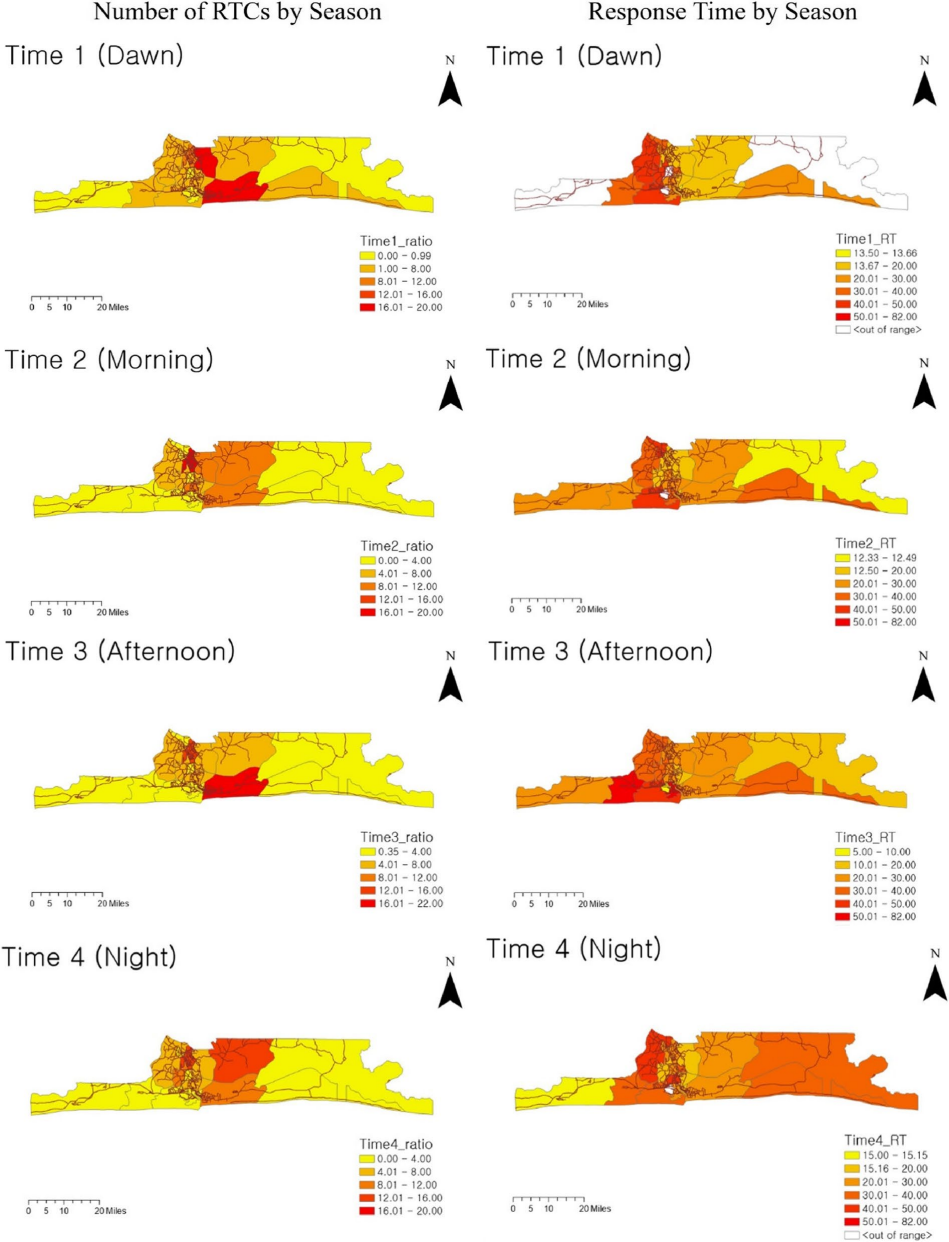
Number of RTCs and response time (RT) by time period in Lagos LGAs

Figure 4 displays the road traffic crashes (RTCs) ratios (left column) and response times (right column) of each LGA by period. The five main LGAs (Eti-Osa, Ikeja, Kosofe, Ikorodu, and Alimosho) areas showed a high number of RTCs during commuting times (Morning and Afternoon). Ikeja, in particular, had consistently high RTC rates throughout the day, regardless of the time period. However, the difference in RTC patterns across time periods was not significant. Conversely, when comparing regional response time patterns, distinct variations emerged for each period. Urban areas such as Alimosho, Amuwo-Odofin, Surulere, and Mainland had longer response times. Notably, Ojo had longer response times during work hours, while Epe had longer response times in the evening. Therefore, timely ambulance deployment adjustments or resource redistribution must be considered. To understand the reason for the response time differences between regions by season and time, this study examined the causes of delay by season and time. This approach helps identify the factors causing disparities between regions and delays in response time.

**Fig. 5:**
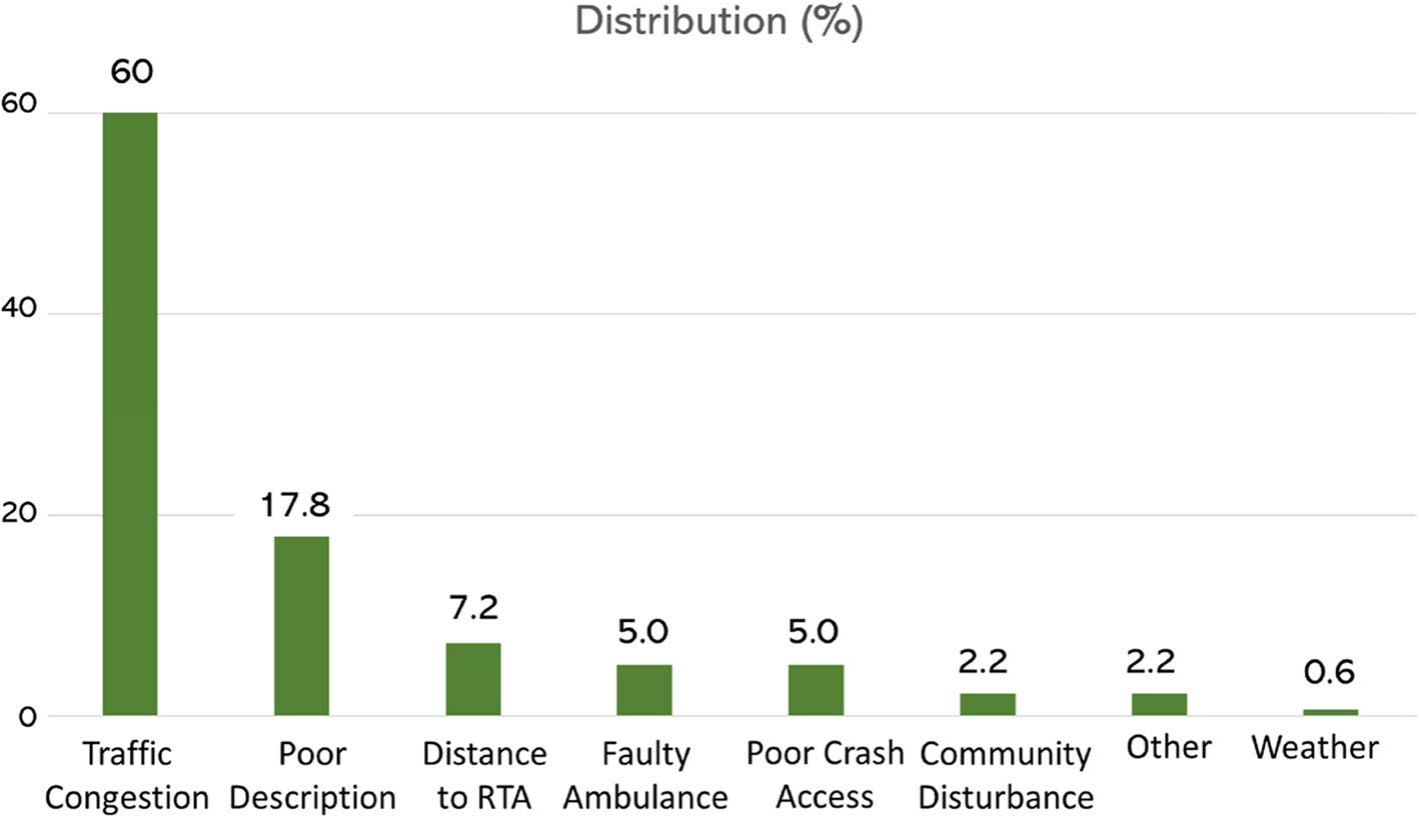
Causes for delayed response

**Fig. 6:**
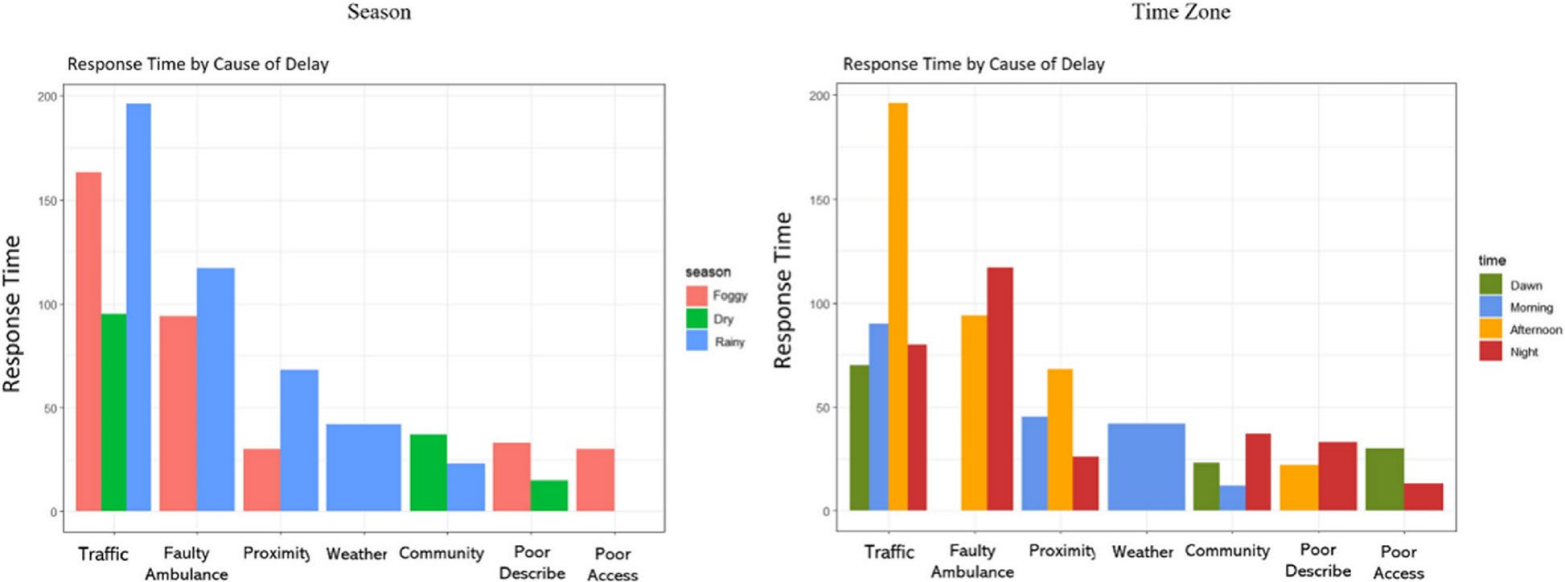
Response time (RT) by cause of delay (season and time period)

Among the Causes for Delay observed in Lagos (Fig. 5), Traffic Congestion was the most frequent (60%), followed by Poor Description (17.8%), and Distance to RTC (7.2%). It shows that the delay is caused by several specific factors. So, we try to understand the distribution of response times by season and time for each Cause for Delay. Figure 6 shows how response times vary for each Cause for Delay. By examining the distribution of response times, we can identify which factors have the greatest impact on response times and prioritize solutions accordingly.

Figure 6 presents response times by season and period for each Cause of Delay. Traffic congestion had the highest response time in all seasons, with the highest response time occurring during the rainy season. Faulty ambulances were the second leading cause of delayed response times, particularly during the Harmattan (foggy) and rainy seasons. The response time for traffic congestion was highest during the afternoon, whereas the response time for faulty ambulances was highest during the night. The results of the pairwise correlation analysis between seasonal response times and Causes for Delay are presented in Table 2 in the Additional file 1 (see footnotes for details). The analysis revealed a statistically significant positive correlation between Traffic Congestion and Response Time in all seasons.

**Table 2:** Descriptive characteristics of recorded RTCs from December 2017 to May 2018

Outcome Indicators / Characteristics	N(%)	Mean	Median	Min	Max
**Participant’s Characteristics**					
**Age** (years)	381 (31.2%)	37	35	7	85
**Gender (*n* = 421)**					
Male	300 (71.3%)				
Female	121 (28.7%)				
**Response Times**					
Time “Call Received” to time “Arrived Crash Scene” (minutes)	1149 (94.1%)	25	20	0	399
**Operation Periods vs. RTC Incidence**					
Diurnal (Day-time) Period 8am – 5 pm	528 (43.3%)				
Nocturnal (Night-time) Period 5 pm – 8am	692 (56.7%)				
**Seasonal Variations vs. RTC Incidence**					
Rainy Season (April, May)	404 (31.1%)				
Harmattan (Fog) Season (December, January)	511(41.9%)				
Dry Season (February, March)	305 (25%)				
**Festive Periods vs. RTC Incidence**					
Christmas Festive Period (December)	195 (16%)				
Ramadan & Easter Festive Period (April)	249 (20.4%)				
**LGA Pop. Density vs. RTC Incidence**					
Urban (Densely Populated) LGAs	674 (55.2%)				
Rural (Sparsely Populated) LGAs	546 (44.8%)				
**Distribution of Outcomes**					
Outcome I: Addressed Crash	449 (36.8%)				
Outcome II: No crash (False Call)	325 (26.6%)				
Outcome III: Crash Already Addressed	270 (22.1%)				
Outcome IV: Did Not Respond	17 (1.4%)				
Outcome V: Other	159 (13%)				

**Fig. 7:**
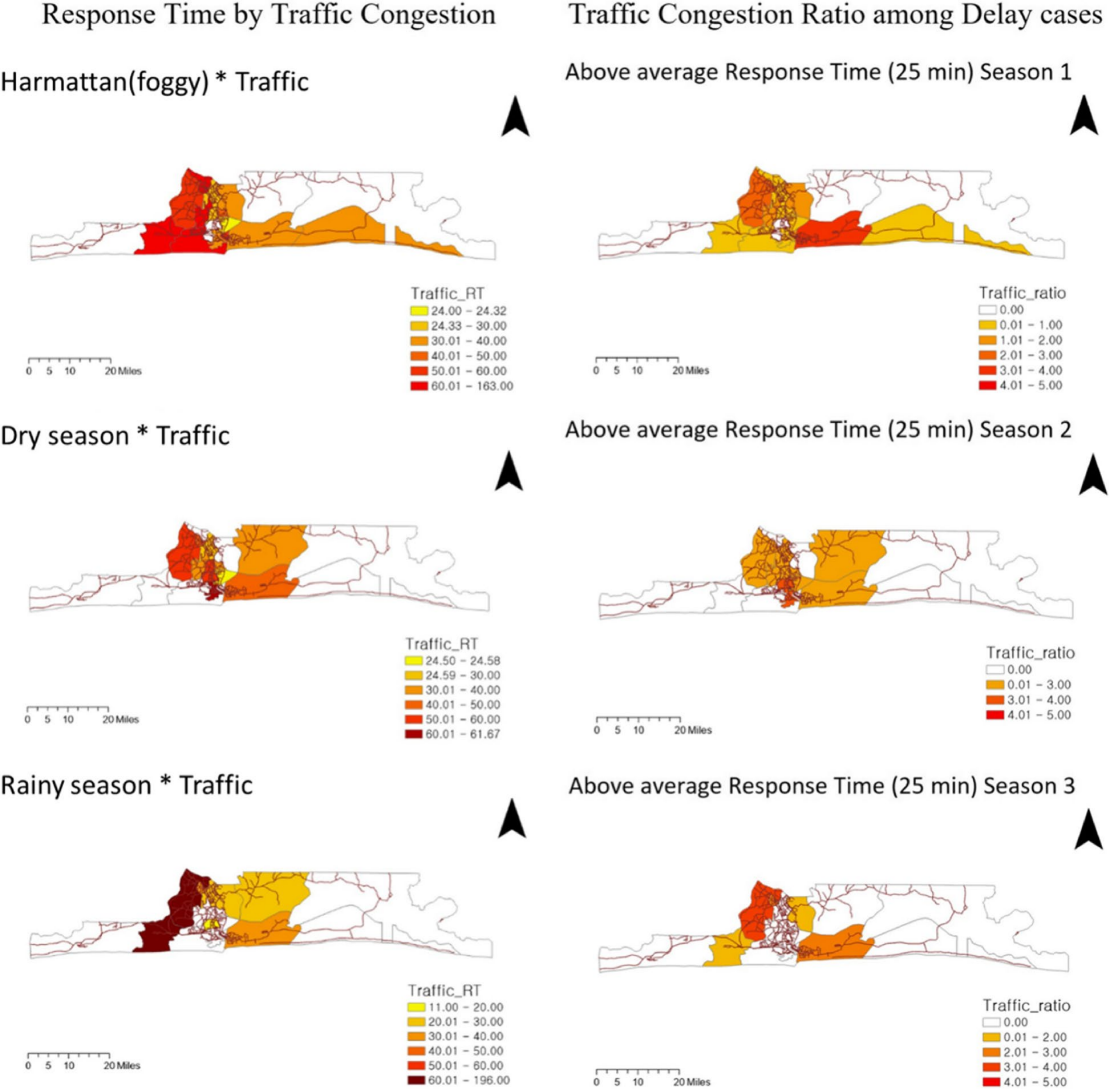
Distribution of response time (RT) by traffic congestion

This finding indicates that traffic congestion consistently delays ambulance response times in Lagos. Therefore, we explored seasonal patterns at the LGA level, focusing on traffic congestion (see Fig. 7). In Season 1 (Harmattan or foggy), the incidence of road traffic crashes (RTCs) due to traffic congestion was highest in the central city center, and the response time in Ojo and Oshodi-Isolo was longer than in other regions due to traffic congestion. In contrast, Mainland and Ikeja had lower response times than other regions. However, the response times in Ojo and Oshodi-Isolo were more than 60 min longer than the average of other regions. In Season 2 (dry), Ikeja had the highest incidence of RTCs due to traffic congestion among the central city centers, and Apapa had the highest response time due to traffic congestion. During the dry season, RTCs due to traffic congestion were highest in the city center, while in the rainy season, they were highest in the sub-center. In Season 3 (rainy), Ojo and Alimosho had the highest response times due to traffic congestion, taking more than 60 min. Ojo had a response time more than 60 min longer than the other regional averages. To address this, seasonal redeployment of ambulance resources or additional resource input from Ojo and Oshodi-Isolo may be necessary.

**Fig. 8:**
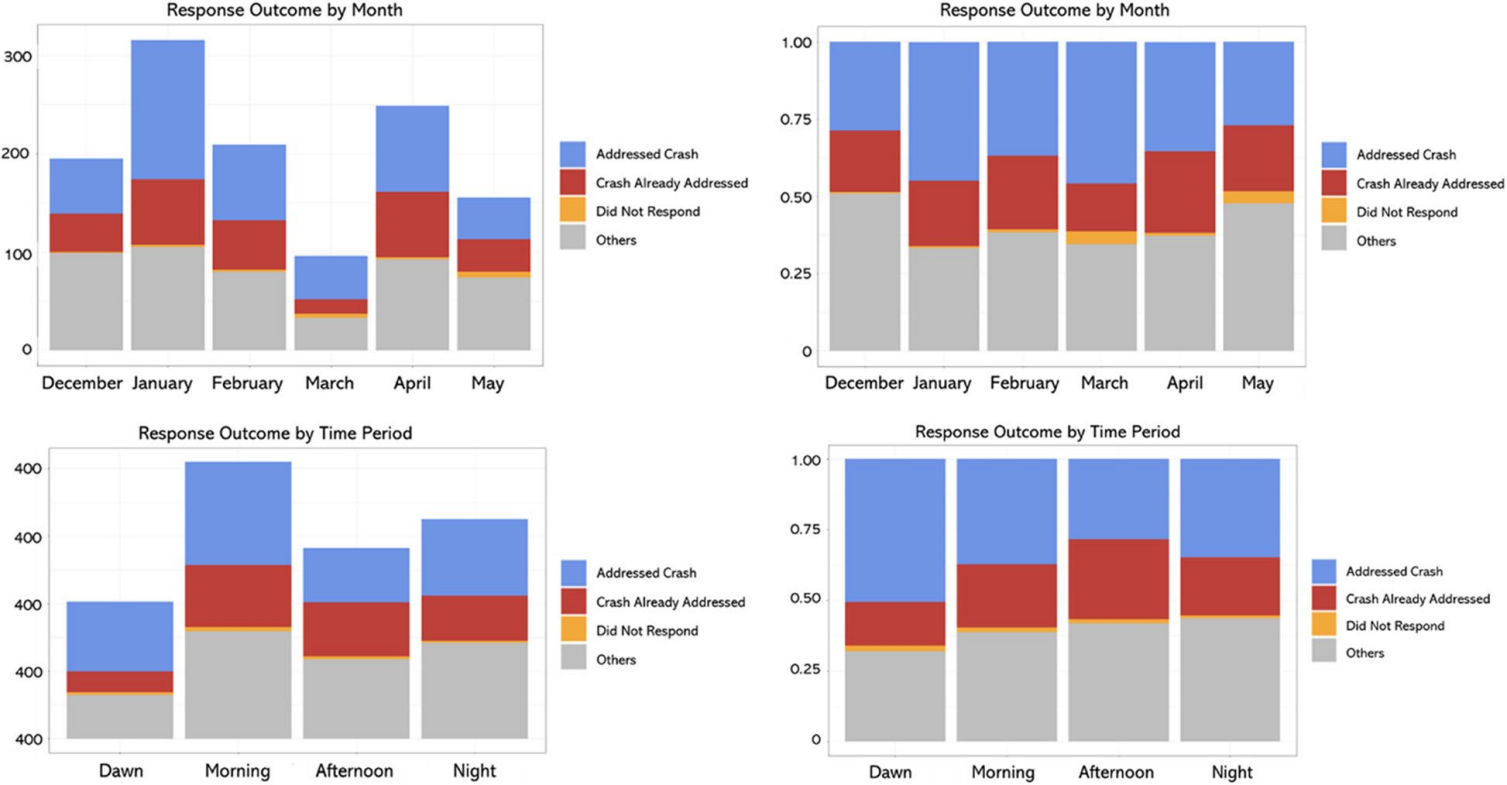
Distribution of main response outcomes

Finally, this study examined the distribution of ambulance response outcomes by season and time. The left column of Fig. 8 shows the frequency of outcomes by season and time, and the right column shows the ratio. We focused on three main categories: Crash Already Addressed, Did Not Respond, and Addressed Crash.

The Main Outcomes examined in this study include Crash Already Addressed, which occurs when an ambulance arrives at an accident scene after delays caused by factors such as traffic congestion, only to find that the accident has already been dealt with. This outcome is crucial to address because it leads to inefficient use of ambulance resources and contributes to social waste. Additionally, the outcome of Did Not Respond indicates a failure to answer an ambulance call, which could have direct consequences for the lives of accident victims. This study aimed to identify characteristics related to ‘Crash Already Addressed’ and ‘Did Not Respond’ among the Outcomes. Examining the distribution of Outcomes by season, the number of Crash Already Addressed incidents was highest in the Harmattan (foggy) season, followed by the Rainy and Dry seasons. Among the Outcomes, Crash Already Addressed occurred at the highest rate during the Rainy season, followed by the Dry and Harmattan (foggy) seasons. By time period, Crash Already Addressed was most frequent in the Morning, and in terms of ratio, it had the highest frequency in the Afternoon, followed by Morning and Night.

**Fig. 9:**
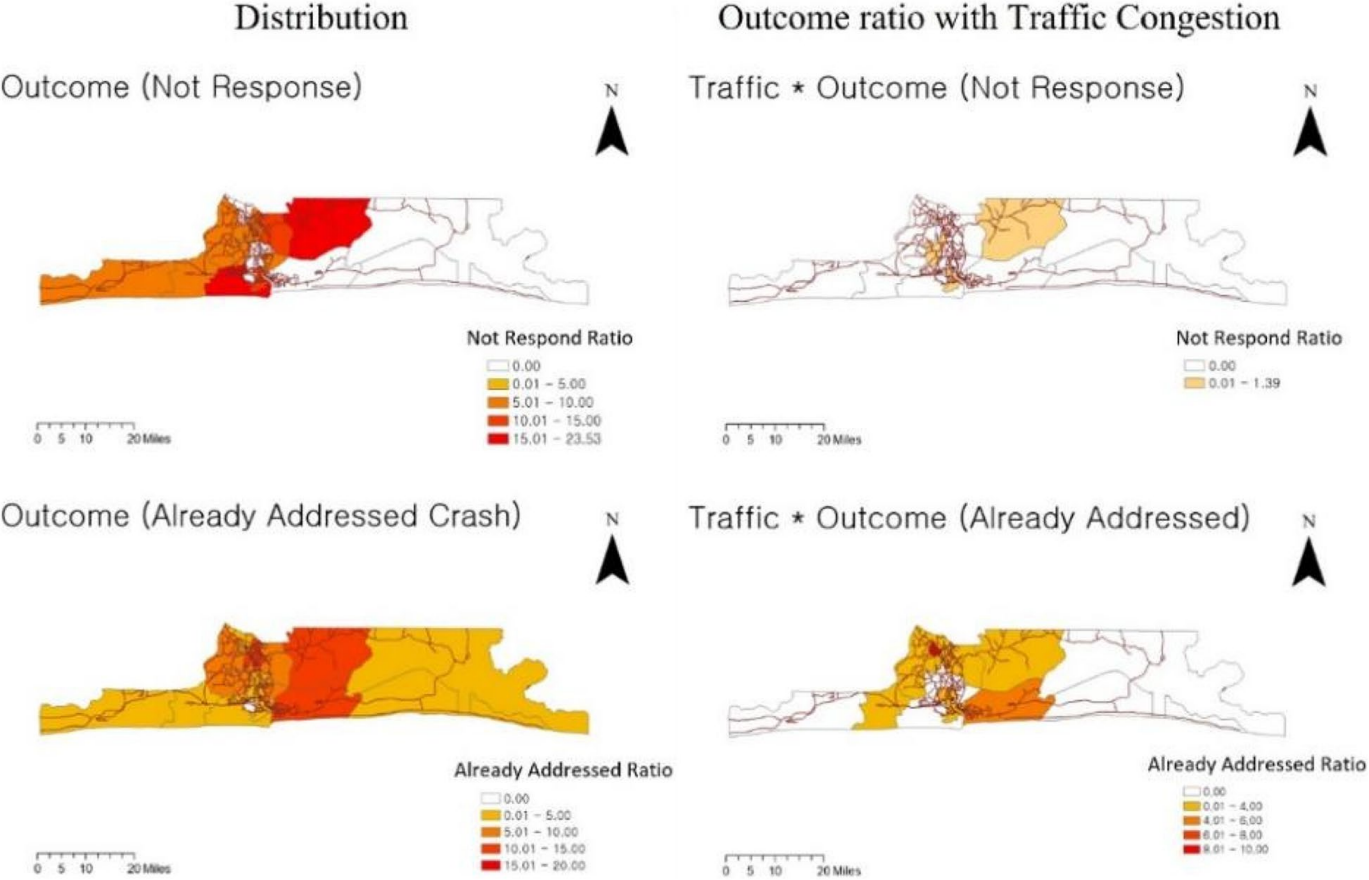
Distribution of RTCs and response time (RT) with outcomes

The white areas on Fig. 9 indicate missing data for Outcomes. Among the response outcomes, Did Not Respond was highest in percentage mainly in the central and western regions, particularly in Ikorodu and Amuwo-Odofin LGAs. Did Not Respond due to traffic congestion was highest in Ikorodu, Oshodi-Isolo, and Apapa, mainly occurring in central and urban areas. Crash Already Addressed by LGA was observed in all regions, with Ikorodu, Eti-Osa, and Ikeja showing the highest rates. Incidents of Crash Already Addressed due to traffic congestion were mainly reported in the central and outlying areas, with Agege having the highest rate compared to other regions, followed by Eti-Osa.

## Discussion

We examined and mapped trends of road crashes and outcomes of pre-hospital trauma care provided by Lagos State Ambulance Service (LASAMBUS) in Lagos state, Nigeria from December 2017 to May 2018. Our goal was to highlight patterns of spatial and temporal characteristics of road traffic crashes (RTCs) and the outcomes of emergency response interventions. We explored determinants and potential for road crashes to occur at particular locations, during particular seasons of a year and within specific time periods of a day. Our data showed that overwhelming majority of those affected were males (73%) which may be related to prevailing traditional male breadwinner cultural practices in Africa. In African societies it is not uncommon to find traditional nuclear families where mostly male partners commute more daily to fend for their families and are therefore at greater risk of RTC compared to their spousal female counterparts. Similar gender distributions in incidences of RTCs have been reported elsewhere [31]. As previously reported [32, 33], and supported by findings from our data, delays in ambulance dispatch and transit following road crashes and distress calls often prolong Emergency Response Times (ERT) and increase potential for fatalities. At the time of this study LASAMBUS had a median ERT record of 17 minutes [24] which it planned to reduce to 12 min in the near future.

The patterns of road crashes in this study followed three dimensions: the circadian dimension revealed temporal relationship of road crashes to distinct time periods in a day with deferring tendencies for crashes to occur at dawn, morning, afternoon or night; the seasonality dimension revealed temporal relationship of road crashes with seasons and disposition for crashes to occur during dry, foggy or rainy seasons of a year; and the festivity period dimension which revealed temporal relationship of road crashes to dominant religious or cultural festivals and the tendency for crashes to occur during Christian Christmas and Easter celebrations or during Muslim Ramadan and Eid celebrations. It appears that increased traveling and commuting during religious celebrations and festivities contributed to road crashes recorded by LASAMBUS, which peaked during the months of April and December. This may imply that religious and cultural festivities have relationship with significant traveling within and across the state and it appears to influence RTCs trends and incidences as many crashes were attended to during Christmas celebration in December (15.5%), and Easter and Ramadan celebrations in April (19.6%). In a reflection of the importance of time period to the crashes, most of the affected locations in our study recorded consistently high numbers of RTCs during peak commuting times (morning and late afternoon). Perhaps the densely populated characteristic of Lagos also contributed to observation that majority of road crash interventions were recorded during peak movement hours of early morning rush to workplaces and early evening rush back home during typical day-time shifts. Conversely the late evening to overnight operation shifts recorded substantially fewer incidences of road crashes since there are comparatively much less human and vehicular traffics then. These observations are congruent with findings from other studies [34, 35] that reported day-time peaks in incidences of road crashes and associated mortality relative to fewer incidences of crash during off-peak periods. A previous study on temporal variations in emergency call responses similarly highlighted influence of time periods ‘time of the day’ and ‘day of the week’ when it reported peak crash incidences at 10am and 7 pm for all emergency and trauma related calls on Friday and Saturday nights [34]. The influence of weather and climate on variations in seasonal incidences of road crashes was also manifest in our data. We found that most road crashes occurred in the harmattan (foggy) season of December and January, and during rainy season month of May, compared to lower incidences recorded during dry season months of February and March. It is reasonable to link this observation to potentially hazardous driving often caused by restricted road access during heavy flooding from rains, and poor visibility associated with cloudy harmattan hazes during these seasons. In line with similar seasonal trend of road traffic crashes (RTCs), researchers from British Columbia, Canada found that higher winter temperature was associated with lower rates of overall RTCs and total fatalities [21].

The trend in distribution of specific locations or hotspots of road crashes appears to be driven by population density of the crash areas. We found that urban densely populated LGAs were popular hotspots for road crashes relative to the rural sparsely populated LGAs. In reality many of the urban LGAs in this study are characteristically overcrowded and tended to have heavy human and vehicular traffics on poorly accessible roads, made worse by inefficient traffic management by local authorities. To mitigate such deficiencies, authorities should implement efficient trauma care plan including judicious reallocation of scarcely available Emergency Medical Service (EMS) resources to known and potential RTC hotspots during peak commuting times and accident prone seasons. Similar pragmatic resource reallocation strategy has been deployed successfully to address pre-hospital trauma care access inequalities more efficiently in accident prone hotspots and neediest areas [14, 36]. In similar vein between 2005 and 2010, a Florida, USA study deployed geospatial analysis technique to identify downtown Miami Dade and South Beach areas of Florida as hotspots for road crashes. Following this, the researchers advised the County to target identified high-risk areas in future planning to reduce road crashes and associated fatalities [22].

Delays which often prolong response times were commonly encountered by LASAMBUS during EMS response operations and trauma care interventions. Among others we found that three outcome variables resulted from some ‘Causes of Delays’, including: Addressed Crash, No Crash (False Calls), and Crash Already Addressed at respectively 36.8%, 26.6% and 22.1% of total interventions. The fact that LASAMBUS was only able to address crashes effectively in just 449 cases representing 36.8% of 1220 response sorties was surprising and suggests that operational constraints could have been encountered by the Emergency Service. Several False Calls for which no crash was found on arrival at crash sites also featured prominently as 325 (26.6%) cases, causing avoidable wastage of scarcely available EMS logistic resources. LASAMBUS personnel opined that the rate of False Calls was high because some members of the public intentionally trigger such calls ‘deceitfully’, purportedly to test sincerity and promptness of the Emergency Service in responding to emergencies. In the past, in efforts to forestall and limit similar public abuses of emergency response calls, an Israeli Study once assessed the effectiveness of a Call-Tracking Technology that had potential to identify and minimize undue harassment calls [37].

In the current study, delayed responses could also have resulted from undesirable interruptions in communications between the Public Caller, the Call Center dispatcher unit and the stand-by Ambulance Unit. This may have resulted from poor description of specific crash location to the Call Center by the public caller. A study once observed that public callers lacked knowledge about the best utilization of emergency number and medical dispatching process and suggested public awareness campaigns to address such shortcomings [38]. Our data also suggests there is a potential for logistic and physical constraints possibly encountered by LASAMBUS in driving through poorly accessible and traffic congested access roads during emergency responses to have contributed to the high level (22%) of ‘Crash Already Addressed’ outcome of total interventions. Perhaps due to poor public perceptions about nature of emergency services, ambulance drivers sometimes have to struggle and contend with other motorists for right-of-way despite blaring emergency sirens. This kind of attitude underscores the urgent need for public reorientation on critical importance of giving priority access to emergency ambulances. Indeed, such public attitude was previously reported in an Iranian study prompting researchers to canvass urgent need for public cooperation to address such attitude and resulting constraints [39]. In this study, the fact that both Traffic Congestion and Poor Access were associated with ‘Crash already addressed’ as a Cause of Delay is attributable to the difficulties LASAMBUS may have encountered in accessing crash sites. In a previous survey involving three years of observation, LASAMBUS was only able to ‘Address Crash’ in a mere 2.3% of 23,537 cases seen at the surgical emergency room of Lagos State University Teaching Hospital [40]. Finally, our data also revealed that LASAMBUS recorded longer emergency response times and higher incidences of RTCs in some densely populated urban LGAs relative to others as a result of traffic congestions on specific key commuting roads during peak commuting hours.

### Limitations

1. Even though we adopted ingenious analytic techniques to eliminate most errors found we were nonetheless constrained to an extent, knowing that our results could have been strengthened further but for the discovery that some of the intervention forms (primary source of our data) showed varying degrees of missing information, supposedly as a result of having gone through error prone, manual (paper) completion process by crews during response transits.
2. Furthermore, being entirely focused on state-organized PTCS the study findings may have limited generalization for leaving out information from the less organized private health sector emergency services where substantial pre-hospital trauma interventions also take place. For a balanced view, future efforts should consider exploring similar geospatial information from the private sector.

## Conclusion

The increasing burden of road traffic injuries in Nigeria is a major public health challenge. Access to quality pre-hospital trauma care services is substantially constrained by limited availability of human and material EMS resources. Pragmatic and innovative approaches may help overcome identified constraints. We explored and highlighted spatial and temporal trends of past road crashes in Lagos State and identified operational constraints encountered by Lagos State Ambulance Service. Our results showed that several factors prominent among which are traffic congestion, poor road access and operational constraints like faulty ambulances negatively impact outcomes of pre-hospital trauma care services provided during peak commuting hours of a day and at specific locations and seasons. The findings offer actionable information that can guide programmatic improvements in pre-hospital trauma care services and limit burden of road traffic injuries in Lagos State. The findings can similarly inform judicious resource allocation and efficient pre-hospital trauma care management and planning to address endemic resource inadequacy and increase population access to pre-hospital trauma care services.

### Supplementary Information

**Additional file 1. **

## Data Availability

The datasets used and/or analyzed during the current study are available from the corresponding author on reasonable request.

## Abbreviations

EMS: Emergency Medical Service
ERT: Emergency Response Time
LASAMBUS: Lagos State Ambulance Service
LCDA: Local Council Development Area
LGA: Local Government Area
LMIC: Low- and Middle Income Countries
PTCS: Post-traumatic Trauma Care Services
RT: Response Time
RTA: Road Traffic Accident
RTC: Road Traffic Crash
RTI: Road Traffic Injury
WHO: World Health Organization
