# Supplementary material for: Spatial and temporal analysis of road traffic crashes and ambulance responses in Lagos state, Nigeria
Source: BMC Public Health. 2023 Nov 17;23:2273. doi: 10.1186/s12889-023-16996-8 (PMC10656774; doi:10.1186/s12889-023-16996-8)
Supplement: Supplementary file 1 — Additional file 1. [file 12889_2023_16996_MOESM1_ESM.docx]

**Additional file 1**

**Table 1. Results of pairwise correlation between Response Time and Cause of Delay**

| Harmattan (foggy) Season | | | | | | | | | | | | | | | | | | | |
| --- | --- | --- | --- | --- | --- | --- | --- | --- | --- | --- | --- | --- | --- | --- | --- | --- | --- | --- | --- |
|  | Response Time | | Poor Access | | Traffic Congestion | | Community | | Weather | | Poor Describe | | Proximity | | Faulty Ambulance | | other | |  |
| Response Time | 1 | |  | |  | |  | |  | |  | |  | |  | |  | |  |
| Poor Access | -0.0368 | | 1 | |  | |  | |  | |  | |  | |  | |  | |  |
| Traffic Congestion | 0.3654* | | -0.0237 | | 1 | |  | |  | |  | |  | |  | |  | |  |
| Community | 00.000 | | 00.000 | | 00.000 | | 1 | |  | |  | |  | |  | |  | |  |
| Weather | 00.000 | | 00.000 | | 00.000 | | 00.000 | | 1 | |  | |  | |  | |  | |  |
| Poor Describe | -0.0417 | | 0.4946* | | 0.0239 | | 0.000 | | 00.000 | | 1 | |  | |  | |  | |  |
| Proximity | -0.01 | | -0.0088 | | 0.0532 | | 00.000 | | 00.000 | | -0.0133 | | 1 | |  | |  | |  |
| Faulty Ambulance | 0.0626 | | -0.0105 | | 0.2388* | | 00.000 | | 00.000 | | -0.0158 | | 0.1593* | | 1 | |  | |  |
| other | 0.395* | | -0.0056 | | -0.0167 | | 00.000 | | 00.000 | | -0.0084 | | -0.0062 | | 0.2623* | | 1 | |  |
| Dry Season | | | | | | | | | | | | | | | | | | | |
|  | | Response Time | | Poor Access | | Traffic Congestion | | Community | | Weather | | Poor Describe | | Proximity | | Faulty Ambulance | | other |  |
| Response Time | | 1 | |  | |  | |  | |  | |  | |  | |  | |  |  |
| Poor Access | | 0.1285* | | 1 | |  | |  | |  | |  | |  | |  | |  |  |
| Traffic Congestion | | 0.1880* | | 0.1942* | | 1 | |  | |  | |  | |  | |  | |  |  |
| Community | | -0.0003 | | -0.0081 | | -0.0263 | | 1 | |  | |  | |  | |  | |  |  |
| Weather | | 0.000 | | 0.000 | | 0.000 | | 0.000 | | 1 | |  | |  | |  | |  |  |
| Poor Describe | | -0.0466 | | -0.0081 | | -0.0263 | | -0.0066 | | 00.000 | | 1 | |  | |  | |  |  |
| Proximity | | 00.000 | | 00.000 | | 00.000 | | 00.000 | | 00.000 | | 00.000 | | 1 | |  | |  |  |
| Faulty Ambulance | | 00.000 | | 00.000 | | 00.000 | | 00.000 | | 00.000 | | 00.000 | | 00.000 | | 1 | |  |  |
| other | | 0.0345 | | -0.0081 | | -0.0263 | | -0.0066 | | 00.000 | | -0.0066 | | 00.000 | | 00.000 | | 1 |  |
| Rainy Season | | | | | | | | | | | | | | | | | | | |
|  | | Response Time | | Poor Access | | Traffic Congestion | | Community | | Weather | | Poor Describe | | Proximity | | Faulty Ambulance | | other |  |
| Response Time | | 1 | |  | |  | |  | |  | |  | |  | |  | |  |  |
| Poor Access | | 00.000 | | 1 | |  | |  | |  | |  | |  | |  | |  |  |
| Traffic Congestion | | 0.3042* | | 0.1670* | | 1 | |  | |  | |  | |  | |  | |  |  |
| Community | | -0.0038 | | -0.0025 | | -0.0149 | | 1 | |  | |  | |  | |  | |  |  |
| Weather | | 0.0429 | | -0.0025 | | -0.0149 | | -0.0025 | | 1 | |  | |  | |  | |  |  |
| Poor Describe | | 0.0852 | | -0.0035 | | 0.2365* | | -0.0035 | | -0.0035 | | 1 | |  | |  | |  |  |
| Proximity | | 0.1621* | | -0.0071 | | 0.1522* | | -0.0071 | | -0.0071 | | 0.2431* | | 1 | |  | |  |  |
| Faulty Ambulance | | 0.1183* | | -0.0035 | | -0.021 | | -0.0035 | | -0.0035 | | -0.005 | | -0.01 | | 1 | |  |  |
| other | | 00.000 | | 00.000 | | 00.000 | | 00.000 | | 00.000 | | 00.000 | | 00.000 | | 00.000 | | 1 |  |
